# Progress in Genetic Studies of Tourette’s Syndrome

**DOI:** 10.3390/brainsci7100134

**Published:** 2017-10-20

**Authors:** Yanjie Qi, Yi Zheng, Zhanjiang Li, Lan Xiong

**Affiliations:** 1 Laboratoire de Neurogénétique, Centre de Recherche, Institut Universitaire en Santé Mentale de Montréal, Montreal, QC H1N 3V2, Canada; qijie416@126.com; 2 Beijing Key Laboratory of Mental Disorders, Beijing Anding Hospital, Capital Medical University, Beijing 100088, China; yizheng@ccmu.edu.cn (Y.Z.); lizhanjiang1221@sina.com (Z.L.); 3 Center of Schizophrenia, Beijing Institute for Brain Disorders, Beijing 100088, China; 4 Département de Psychiatrie, Faculté de Médecine, Université de Montréal, Montreal, QC H3C 3J7, Canada; 5 Department of Neurology and Neurosurgery, McGill University, Montreal, QC H3A 2B4, Canada

**Keywords:** Tourette Syndrome, gene mapping, genetic association, genetic linkage, complex trait, heritability, epigenetic, genome-wide association study, whole exome sequencing, whole genome sequencing

## Abstract

Tourette’s Syndrome (TS) is a complex disorder characterized by repetitive, sudden, and involuntary movements or vocalizations, called tics. Tics usually appear in childhood, and their severity varies over time. In addition to frequent tics, people with TS are at risk for associated problems including attention deficit hyperactivity disorder (ADHD), obsessive-compulsive disorder (OCD), anxiety, depression, and problems with sleep. TS occurs in most populations and ethnic groups worldwide, and it is more common in males than in females. Previous family and twin studies have shown that the majority of cases of TS are inherited. TS was previously thought to have an autosomal dominant pattern of inheritance. However, several decades of research have shown that this is unlikely the case. Instead TS most likely results from a variety of genetic and environmental factors, not changes in a single gene. In the past decade, there has been a rapid development of innovative genetic technologies and methodologies, as well as significant progresses in genetic studies of psychiatric disorders. In this review, we will briefly summarize previous genetic epidemiological studies of TS and related disorders. We will also review previous genetic studies based on genome-wide linkage analyses and candidate gene association studies to comment on problems of previous methodological and strategic issues. Our main purpose for this review will be to summarize the new genetic discoveries of TS based on novel genetic methods and strategies, such as genome-wide association studies (GWASs), whole exome sequencing (WES) and whole genome sequencing (WGS). We will also compare the new genetic discoveries of TS with other major psychiatric disorders in order to understand the current status of TS genetics and its relationship with other psychiatric disorders.

## 1. Introduction and Genetic Epidemiology of Tourette’s Syndrome

### 1.1. Clinical Features of Tourette’s Syndrome

Tourette’s Syndrome (TS) is a complex neuropsychiatric and developmental disorder characterized by repetitive, sudden, involuntary movements and vocalizations, called tics. Vocal tics demonstrated as the involuntary outburst of obscene words, or socially inappropriate and derogatory remarks, called coprolalia, are rare (in about 8% of TS patients) according to the Diagnostic and Statistical Manual of Mental Disorders, Fifth Edition (DSM-5) [1]. TS is the most serious form of a spectrum of tic disorders [1], not only due to its complexity and duration of tic symptoms, but also due to its higher comorbidity, impairment of social function and compromised quality of life [2]. In TS, tics typically start at 4–6 years old; and the severity peaks around 10–12 years old [3]. The course of TS waxes and wanes [4]. Tic symptoms increase during times of stress, anxiety, fatigue and decrease with focused concentration, such as processing fine motor movements [5]. For long-term clinical course, tic symptoms gradually ease during adolescence. Until early adulthood, approximately 75% of patients have a different degree of decreased tic symptoms, including one-third of patients with complete remission; but a quarter of patients continue to have tics in adulthood [3]_,_ which has been shown to be correlated with the severity of tics in late childhood [6,7]. Studies have also shown that comorbidities presented at the onset showed a more severe prognosis [6,8,9]. In addition, poor fine-motor skills and smaller caudate volume in childhood were found to be associated with increased adulthood tic severity [10,11]. These may represent different subtypes of TS, and need to be taken into consideration when planning genetic studies to identify the underlying causes.

### 1.2. Comorbidities of TS

As one of the prominent characteristics compared to other neuropsychiatric disorders, TS is most often associated with other mental and behavioral symptoms and psychiatric diagnoses [12,13], such as obsessions, compulsions, hyperactivity, distractibility, and impulsivity. Studies have shown that 88–92% of TS patients also suffer from one or more comorbidities, such as attention deficit hyperactivity disorder (ADHD), obsessive-compulsive disorder (OCD), Non-OCD anxiety disorders, mood disorders, disruptive behavior disorders, pervasive developmental disorders and problems with sleep [12,14,15,16]. A significant subset of children with TS also have fine motor control and visual-motor integration impairment. The most common age for comorbidity occurrence is around 4–10 years old, except for eating disorder and substance use disorder, which usually begin around 15–19 years old [12]. The most common comorbidity of TS is ADHD, ranging from 60–80%; the second common comorbidity is OCD, ranging from 11–80%, other comorbidities are rage attacks (25–75%), depression (13–76%), sleep problems (12–62%), migraine (25%), and learning disabilities (23%) [13]. High frequencies of comorbidities of TS indicate its clinical heterogeneity, as well as heterogeneity and complexity of the underlying etiologies, pathogenic pathways and neural networks.

### 1.3. Prevalence of TS

Several systematic reviews, in a total of 44 population studies from Europe, East Asia, Middle East, Far East, Oceania and North America, have reported that TS prevalence is between 0.3% and 0.9% in children [17,18,19]. TS has been reported in different cultures across different populations worldwide with similar phenomenology, but with lower prevalence in the Afro-Americans in the USA and the sub-Saharan Black Africans [17]. There were fewer studies on the prevalence of TS in adults. The prevalence of TS in adults was estimated between 0.05% and 0.1% [19,20]. In general, tic disorders are less common in adults than children, which is consistent with partial or complete remission of tic symptoms during and after adolescence. In addition, studies have also shown that TS prevalence is higher in males than females. Male/female ratio is about 3–4:1 [19,21]. The latest study involving 122,884 Canadians suggested that prevalence of TS is 0.33% and 0.07% in youth (12–17 years old) and adults (>18 years old), respectively; and the sex ratio is reported to be 5.31:1 in youth, and 1.93:1 in adults in this study [22]. Schlander et al. also described that the difference of TS prevalence between males and females decreased in adults [23]. Therefore, TS is a typical neurodevelopmental disorder, which is also influenced by sex difference during brain development.

### 1.4. Heritability of TS

Family studies have shown that the rate of TS in first-degree relatives of TS patients is 10-to-100-fold higher than general population [24]. The similar results (7- to 22-fold increase) were found in first-degree relatives of patients with chronic tic disorders (CTDs) [24]. In family studies, the heritability of tic disorders was estimated to be between 0.25 and 0.77 [20,25]. The results from the latest family study, which included 4826 individuals with TS or CTDs, suggested that the rate of TS or CTD in first-degree relatives was higher (odds ratio, OR: 18.69) than in second-degree relatives (OR: 4.58) and third-degree relatives (OR: 3.07), when compared with unrelated controls (matched on a 1:10 ratio) [25].

There were also family studies regarding the co-heritability of TS and its comorbidities. In a study of 222 affected sibling-pair (ASP) families collected by the Tourette’s Syndrome Association International Consortium on Genetics (TSAICG), TS, OCD, ADHD, and ADHD + OCD were all found highly heritable in this TS-enriched family cohort; and a significant familial correlation was found between TS and OCD, and between OCD and ADHD. However, there was no significant familial correlation between TS and ADHD; whereas they seemed to share significant environmental correlation [26]. Another study of TS and ADHD, not OCD, suggested that TS and ADHD may still have some overlapped genetic factors [27].

Although initially some single large pedigrees of TS suggested autosomal dominant inheritance [28,29,30,31,32], further complex segregation analysis and principal component analysis in extended large pedigrees ascertained through TS probands indicated that the pattern of TS and other related tic disorders was not consistent with simple Mendelian inheritance, even after modelling explanatory variables, such as obsessive compulsive symptomatology [33]. Notably, there seem to be significant phenotypic variability within pedigrees in terms of severity and comorbidity [34].

Twin studies are important and valuable approach to delineate the genetic contribution to genetic disorder like TS. Price and his collages studied 43 pairs of same-sex twins, including 30 pairs of monozygotic (MZ) and 13 pairs of dizygotic (DZ) in 1985 [35]. Concordances rates for TS were 53% for MZ and 8% for DZ. For tics, concordance rates were up to 77% and 23% for MZ and DZ pairs, respectively. The subsequent study on 16 pairs of MZ twins showed the concordance rate were 56% for TS and 94% for tic disorders [36]. In 2007, Bolton et al. investigated a community sample involving 4662 6 years old twin pairs using a two-phase design. The heritability was estimated at 0.50 for tic disorders; and by assessing 854 pairs at the second phase, concordance rate for tic disorders was 0.64 for MZ pairs and 0.33 for DZ pairs [37]. Another study screened a cohort of Swedish 9 and 12 years old for multi-psychiatric diseases, including tic disorders, autism spectrum disorders (ASD), ADHD, OCD etc. [38]; a total of 17,220 twins were recruited. The results suggested that genetic correlations accounted 0.56 of heritability (95% CI: 0.37–0.68), with concordance rate of 0.38 for MZ pairs and 0.11 for DZ pairs for tic disorder. The same study also estimated the heritability by sex, i.e., 0.26 in girls and 0.39 in boys [38]. The tic heritability of adult twins was estimated at 0.33 in another study by Pinto et al. in 2016 [39].

Both family and twin studies have shown that the etiology of TS is at least partially of genetic origin; however, the inheritance is more likely to be complex than simple Mendeian mode. In general, TS and associated comorbidities have much lower heritability compared to other neuropsychiatric disorders, such as ASD (heritability: 0.80), schizophrenia (SCZ) (heritability: 0.81) and bipolar disorder (BPD) (heritability: 0.75) [40]., indicating other factors during development and from the environmental also play some roles in pathogenesis of TS and its comorbidities [37,38,39].

## 2. Genome-Wide Linkage Studies of TS

Due to the autosomal dominant-like inheritance pattern in some large extended pedigrees with TS and comorbidities, earlier stage of genetic research of TS had focused on genome-wide linkage studies (GWLSs) of TS families, which assess the probability in a given pedigree or pedigrees, where the disease and the genetic marker(s) are cosegregating. Several chromosomal regions were reported as potential candidate loci for TS through GWLSs, including chromosome 3p21–p14 [41], 4q34–q35 [42,43], 5q35.2–q35.3 [43], 6p21 [44], 7q31 [45,46,47], 11q23–24 [48,49,50], 13q31.1 [51], 15q21.1–15q21.3 [52], and 17q25 [43,53]. However, despite the earlier excitement and optimism about gene discovery for TS through GWLS approach, and some chromosomal regions have been implicated in some TS families, no gene or causal mutation of major effect has been discovered for the above-mentioned TS loci, except for the *SLITRK1* locus on chromosome 13 [51] and the *HDC* locus on chromosome 15 [52]. The reasons behind these earlier disappointing gene discovery endeavors through GWLSs may be multiplicative, including phenotypic complexity, such as clinical and genetic heterogeneity of TS and its associated comorbidities, limited sample size, as well as limitation of the previous genetic technologies and statistical methodologies, e.g., the low coverage and resolution of the microsatellite marker panels. Since linkage analysis is based on calculation of recombination events in limited number of generations, it is sensitive to misspecified genetic information, such as individual affection status, allele frequency, mode and parameters about inheritance. Nevertheless, limited significant statistical results of TS GWLSs strongly suggest that the underlying genetic architecture of TS and associated comorbidities is more complex than simple Mendelian inheritances.

## 3. Candidate Gene Association Studies of TS

After unsuccessful GWLS attempts in TS gene discovery, researchers had turned to direct candidate gene linkage and association studies to identify the potential genetic contribution of TS. Based on progresses in neuroimaging, postmortem, animal models and pharmacological studies, it has been hypothesized that the cortico-striatal-thalamo-cortical (CSTC) pathways may underlay the pathogenesis of TS and related symptomatology; and abnormalities of those neurotransmitters and their signal transmissions could lead to the dysfunction of CSTC neural network and subsequent clinical manifestation of TS. Multiple neurotransmitter systems have been implicated in CSTC pathways [54], including dopaminergic, serotonergic, glutamatergic, gamma-amino butyric acid-(GABAergic), histaminergic, and other neurotransmitters [55,56,57]. Furthermore, a neurotransmitter dysfunction may also cause change in other neurotransmitters due to interaction or self-regulation among them [57].

Compared to genetic linkage analysis, genetic association is when one or more genotypes within a population, i.e., unrelated cases and controls, co-occur with a phenotypic trait more often than would be expected by chance occurrence. In earlier stage of genetic research of TS, many studies have been carried out to test genetic linkage and associations between various neurotransmitters and TS. Table 1 is a summary of candidate gene linkage and association studies of TS. In general, none candidate gene showed positive linkage result. Some association studies of TS showed positive results; however, their effects in TS seemed to be very small; and very often results were non-replicable; sometimes even controversial. As shown in Table 1, most studies were limited by sample sizes, therefore under power according to current standards. Interestingly, since 2005, majority of genetic association studies have achieved positive results, accounting for bigger sample size, more advanced technical methods and selected genes.

## 4. Genome-Wide Association Studies of TS and Other Psychiatric Disorders

Facilitated by the advent of SNP array technologies and statistical methodologies, as well as the collective efforts of large scale biobanks for research materials, genome-wide association study (GWAS) has become the main stream of genetic studies of common diseases in the past decade. The current GWAS Catalog contains 3055 publications and reports 39,360 unique SNP-trait associations in nearly 2000 traits or diseases [95]. Psychiatric disorders fall into the complex trait category; and most psychiatric disorders are common and highly heritable. Therefore, GWAS has been the focus of considerable effort in field of psychiatric genetics in the past decade. To date, over 400 GWAS studies have been published in psychiatric disorders like SCZ (104 GWAS analyses) [95,96,97], ASD (23 GWAS analyses) [95,98,99], BPD (85 GWAS analyses) [95,100,101], MDD (major depressive disorder) (55 GWAS analyses) [95,102,103], OCD (5 GWAS analyses) [95,104,105] and ADHD (33 GWAS analyses) [95,106,107]. These research results have markedly increased our understanding and knowledge of the genetic basis of these psychiatric disorders, and have yielded empirical data on genetic architecture and pathways critical to address the long-standing debates in the field of psychiatry. Table 2 shows a summary of GWASs of several main psychiatric disorders. In general, the effects of risk variants from GWASs are small but wide-spread genome-wide; and the majority of GWAS-identified variants fall in noncoding regions of the human genome. However, further annotations indicate that these regions are enriched for active elements for gene expression regulation in relevant cell functions [108,109].

So far, only two GWASs of TS and one replication study have been published to date; one reached a marginal genomewide significance and the other GWAS failed to identify genome-wide significant loci for TS [110,111,112]. These results may have a close relationship with the sample size (1285 cases/4964 controls, and 2498 cases/6277 controls, respectively for each TS GWAS), which were much smaller than other major psychiatric GWASs. To date, there are no significant finding in ADHD and OCD GWASs either, most likely due to limited sample sizes as well. Nevertheless GWASs of ADHD and OCD found some top signals within some interesting candidate genes, such as *LMOD2*, *WASL*, *ASB15*, and *SHFM1* genes in ADHD GWASs [113,114]; and *PTPRD* [104] and *FAIM2* [105] genes in OCD GWASs.

One unambiguous conclusion from all GWASs is that complex traits are polygenic; and multiple lines of evidence are consistent with widespread pleiotropy for complex traits; these features are particularly striking in GWASs of psychiatric disorders. Widespread but small effects indicate that these common risk variants are not the main cause of disease phenotype under study, but rather just reflect the minimal peripheral polymorphic effects of the underlying molecular networks in different individuals and populations; and in case of TS GWAS, the subtle functional diversity or polymorphisms of the CSTC network.

## 5. Chromosomal Abnormalities and Copy Number Variants (CNVs) of TS

Chromosomal aberrations studies are an important aspect of TS genetics with more significant findings. By single or combined in situ hybridization, chromosomal microarray, cytogenetic and next-generation sequencing, chromosomal aberrations studies have identified several large, rare structural aberrations associated with TS and related phenotypes. Table 3 is a summary of some main candidate loci and genetic findings. Among the most interesting candidate genes, *Slit* and *Trk-like*, *family member 1* (*SLITRK1*) gene on 13q31.1, discovered in a TS patient with a de novo inversion [51]. SLITRK1 is a transmembrane protein that regulates neurite outgrowth by phosphorylation-dependent manner; and it has been shown to control neurite outgrowth; and it is expressed in the embryonic and postnatal brain, including the cortex, thalamus, and basal ganglia, which correlate with the neuroanatomical regions most commonly implicated in TS [119,120,121]. *Inner mitochondrial membrane protein 2L* (*IMMP2L*) gene on 7q22–q31 is also an interesting candidate gene for TS, disrupted by a breakpoint in 7q31 in TS patients, as well as implicated in autism and speech-language disorders [45,46,122,123]. *IMMP2L* encodes the inner membrane peptidase subunit 2, a mitochondrial protease involved in cleaving the space-sorting signals of mitochondrial membrane proteins; and defective IMMP2L may lead to disrupted mitochondria function [123]. Mitochondrial dysfunction has been associated with a range of human disorders, including neuropsychiatric disorders [123]. Disruptions of *Contactin associated protein-like 2* gene (*CNTNAP2*) on 7q35–q36 [124,125] and *Neuroligin 4* (*NLGN4*) on Xp22.3 [126] have also been reported to be associated with TS and other neurodevelopmental disorders.

CNV is a special type of structural variation, i.e., a type of duplication or deletion event that affects a considerable number of base pairs at the same chromosomal location [142]. CNVs have been identified as causal genetic variants in other psychiatric disorders [143,144]. It has been reported that de novo CNVs are strongly associated with ASD and SCZ [143,144,145,146,147,148,149]. In ASD, de novo CNV in simplex families is 5 times and 10 times higher than multiplex families and controls, respectively [145]. In SCZ, de novo CNV is 8 times higher in sporadic cases than in controls [148]. It has been estimated that increased large CNV and chromosomal rearrangement may contribute to 5–10% risk of ASD [149]. The other neurodevelopmental conditions, such as bipolar disorder, epilepsy, and intellectual deficiency also share a highly similar large CNV landscape with potential pathogenicity [150,151].

Table 4 summarizes CNV studies in TS. Approximately 1% of TS cases carry one of these CNVs, indicating that rare structural variation contributes significantly to the genetic architecture of TS structural variants, as well as increased global CNV burden that is mainly driven by large, rare, clinically relevant events, contribute significantly to the genetic architecture of TS, in which larger CNVs could create imbalance for more genes during neurodevelopment and lead to more severe outcomes.

## 6. Whole Exome/Genome Sequencing Studies of TS and Other Psychiatric Disorders

Whole exome sequencing (WES) is a fast and cost-effective method by sequencing all coding regions of the entire human genome to detect rare deleterious variants, which disrupt encoded protein function. Whole genome sequencing (WGS) is to sequence the entire genome, including intron, exon, flanking sequence and interval regions. Unlike GWASs that identify common variants (frequency ≥ 5%), WES/WGS are used to identify rare (frequency ≤ 1%) and novel variants genome-wide. WES has achieved great success in Mendelian disorders [157]; but also used as an effective means to identify loci for complex traits or diseases [158]; and these protein-coding regions contain 85% of disease-causative mutations, even if they only cover less than 2% of the entire genome [159,160,161]. Coming after WES, WGS allows to identify private genetic variants, small insertions and deletions (indels), more complex CNVs, and other structural alterations; and most of these variants reside in the ~98% noncoding genome largely unexplored by SNP microarray and WES studies. With the application of WES/WGS, some rare mutations including de novo point mutations, gene-disrupting structural mutations have been found in psychiatric disorders [162,163,164,165], in which loss of functional variants (nonsense, splice-site variants), and increased burden of de novo mutations, have been shown to play an important role in etiology of psychiatric disorders [165].

Some major studies and findings of WES and WGS in psychiatric disorders are summarized in Table 5. So far, the application of WES has generated the most significant results in ASD than in any other psychiatric disorders, and multiple large scale studies have identified numerous candidate genes for ASD [166,170,171,174,186,187,188,189,190,191,192,193,194,195]. Compared to WES, WGS studies are much newer and with fewer number of studies and with smaller sample sizes for current studies. However, with reduced cost and improved methodologies, the application of WGS has been rapidly increased in genetic studies of complex disorders [179,180,181,196,197,198]. WES/WGS studies have further demonstrated that psychiatric disorders like SCZ, BPD, ASD belong to complex traits, rather than monogenetic disorders; and the underlying pathogenic mechanisms and pathways are perplexing. There are fewer studies of WES/WGS in TS. Sundaram et al. performed WES in ten members of one pedigree with seven affected by TS; and identified three novel nonsynonymous variants within MRPL3, DNAJC13 and OFCC1 genes [199]. Willsey et al. performed WES in two TS cohorts (325 trios from the Tourette International Collaborative Genetics cohort; 186 trios from the Tourette Syndrome Association International Consortium on Genetics), and suggested that de novo damaging variants found in approximately 400 genes may contribute to genetic risk in 12% of clinical cases of TS; and they particularly reported 4 likely risk genes for TS, i.e., WWC1 (WW and C2 domain containing 1), CELSR3 (Cadherin EGF LAG seven-pass G-type receptor 3), NIPBL (Nipped-B-like), and FN1 (fibronectin 1) [200]. Eriguchi et al. identified 30 de novo mutations, including four missense mutations (RICTOR, STRIP2, NEK10, and TNRC6A genes) in WES of nine trio families and one quartet family of TS [201]. Sun et al. identified a nonsense mutation through WES in the PNKD gene segregating with TS and Tic disorders in six out of nine members in a three-generation TD multiplex family [202]. These five above-mentioned candidate genes are enriched in CSTC regions, which are implicated TS; and their expression levels vary in different development stages, suggesting that disruptive protein function of those genes might affect neuronal development or activity in these brain structures [201,202]. Compared to ASD, SCZ and MDD, WES/WGS in TS are limited by the small sample sizes [199,200,201,202]. Further studies with enlarged sample size will be helpful to understand the genetic architecture of TS and its overlap with other neuropsychiatric disorders.

## 7. Epigenetics and TS

Decades of genetic research have shown that there are no direct correlations between genotypes and phenotypes in psychiatric disorders; and there ought to be missing pieces of information between genomic variations and disease phenotypes, i.e., changes of genomes will only become effective through transcsriptome, epigenome and result in phenotypic outcomes. Epigenetics refers to the ongoing regulation of gene expression, which includes structural modification of chromatin, post-translational modification of histones (i.e., acetylation and methylation), chemical modification of DNA through methylation or hydroxymethylation of cysteins, as well as expression of interfering non-coding RNAs (i.e., miRNAs and long-non-coding RNAs) [203,204]. These epigenetic mechanisms allow reprogramming of the genome upon environmental inputs at specific time-points during development and through lifetime. The epigenetic changes triggered by early life events stand as a valuable hypothesis of the environmentally induced behavioral changes.

Epigenetic regulation has been shown to have an impact on the development of many neuropsychiatric disorders [205,206], and to play a pivotal role in embryonic and adult neurogenesis [207]. Some studies on epigenetic processes in the expression and inheritance of behaviors have helped us to further understand the complexity of brain functions [208]. Abelson et al. identified a noncoding RNA variant (var321) at the binding site for microRNA hsa-miR-189 in two unrelated TS patients on chromosomal 13 [51] (see above). In 2015, Rizzo et al. found that miR-429 was significantly underexpressed in TS patients and suggested as an useful molecular biomarker to aid TS diagnosis in the future [209]. Another study suggested that methylation levels of the *DRD2* gene were higher in adults with TS than in sex-, age-matched controls [210]; and methylation of *DRD2* was positively correlated with tic severity. However, methylation of *DAT* was negatively correlated with tic severity in the same study [210]. State et al. reported that there might be relationship between chromosome 18(q21–q22) inversion and epigenetic mechanism in a 12 year old boy with TS and OCD [140]. The results from the first epigenome-wide association study, which investigated DNA methylation in patients with tic disorders, suggested that the top hits of methylation signal were enriched for genes involved in brain-specific and developmental processes [211]. Delgado et al. did not find significant methylation difference in TS patients compared with controls in a region of chromosome 8 for *KCNK9* and *TRAPPC9* genes [212].

## 8. Conclusions

TS is a complex disorder with highly variable phenotypic manifestations and high frequency of comorbidities. It is more common in males than in females. Family and twin studies have shown that genetic factors play an important role in the pathogenesis of TS. Candidate genes studies have shown that multiple genes (*DRD2*, *DRD4*, *5-HT2C*, *SERT*) in multiple neural systems, including dopaminergic, serotonergic, histaminergic pathways, might be associated with pathogenesis of TS, but results are not yet convincing enough. In recent years, several new candidate genes, e.g., *SLITRK1*, *IMMP2L*, *CNTNAP2*, *NLGN4*, have been identified through linkage studies and structural genomic aberrations, in which very rare genetic variants with large effects were found in TS patients and families.

Compared to genetic studies of other psychiatric disorders, TS studies are currently still largely limited by the relatively small sample sizes, because of the complexity and heterogeneity in clinical manifestation. We expect that more rare and common variants with various effects underlying the genetic susceptibility of TS will be discovered through larger-scale international collaborative projects in the near future. Combinational analyses of common variants with rare variants, structural variations and CNVs, plus epigenetic factors; i.e., integrative “multi-omics” together with environmental factor analyses [213,214] will be the future direction. As promising starts, four large collaborative groups with joint effort and multiple resources have been developed for TS genetic research, focusing on existing large well-characterized patient cohorts, which include (1) the Tourette Syndrome Association International Consortium for Genetics (TSAICG) [215]—genomewide association studies for TS; (2) the Tourette international collaborative genetics (TIC genetics) study—whole exome sequencing in families with TS [216], which is funded by the National Institute of Mental Health (NIMH) in the USA; (3) the European multicentre tics in children (EMTICS) study—exploring gene-environment interactions that underlie TS etiology, which is funded by the European Commission under the Seventh Framework Programme, including 17 clinical sites from across Europe [217]; (4) TS-EUROTRAIN—coordinating large-scale studies and training the next generation of experts for TS, supported by the European Commission [218,219]; (5) Of particular interest in this field, the PsychENCODE project has been created and developed by an international consortium to provide an enhanced framework of regulatory genomic elements (promoters, enhancers, silencers and insulators), catalog epigenetic modifications and quantify coding and non-coding RNA and protein expression in tissue and cell-type-specific samples from healthy (neurotypical) control and disease-affected post-mortem human brains, as well as functionally characterize disease-associated regulatory elements and variants in model systems. [220]. The Project is currently focusing on three major psychiatric disorders: ASD, BPD and SCZ, for example, 98 of the 108 independently associated loci were found in non-coding region in SCZ GWAS [96,221] suggesting that the PsychENCODE project will be a valuable platform to study the epigenetics of psychiatric disorders in the future, including TS studies, in which some preliminary results indicated that epigenetics might play a role in TS as well [51,140,209,210,211].

In the near future, GWAS by SNP arrays will most likely be gradually replaced by WGS, with increased power by including rare variants in the genetic analyses. For many years, genotyping technology was the limiting step to genetic discoveries, but now discovery is limited by phenotypic descriptors that could link with genetic data to allow disease stratification, which might be more aligned with treatments; therefore, deep phenotyping aligned with genetic studies will also facilitate new discoveries for disease risk and mechanism.

Although with limited results, epigenetic research has shown promising hope to link the genomic variations with environmental exposures and disease outcomes, particularly for psychiatric disorders and behavioral phenotypes. For TS, further studies with larger sample size could focus on further understanding of the effect of dynamic epigenome on the regulation of developmental genes and behaviors. Additional large-scale studies could also aim to disentangle common versus disorder-specific genomic and epigenomic variations in TS with other psychiatric and behavioral phenotypes. Understanding the mechanism behind the sex differences in TS may also help us better understand the regulation of gene expression in the brain and its implication in TS pathogenesis because TS is a male-biased disease.

## Figures and Tables

**Table 1:** Main findings of linkage and association studies of neurotransmitter genes in Tourette’s Syndrome (TS).

Gene	Chromosome	Finding	Sample Size (Ethnicity)	Reference
**Dopaminergic**				
**DRD1** (Dopamine receptor D1)	5q34–35	No association was found between DRD1 and TS in both family study and case-controls studies. (LOD < −2)	One pedigree, 85 interviewed in 116 members (NA *)	[58]
			One large pedigree (Mennonite)	[59]
			50 TS, 35 TS + ADHD, 30 TS + OCD, 50 controls (NA)	[60]
			148 TS and 83 controls (Taiwanese)	[61]
**DRD2** (Dopamine receptor D2)	11q22–23	DRD2 (TaqIA) was associated with tic severity and overlapped with other psychiatric disorders; multiple SNPs of DRD2 ((H313H) C) were associated with TS. (*p* = 0.001–0.03)	147 TS and 314 controls (Non-Hispanic Caucasian)	[62]
			225 TS and 67 controls (European)	[63]
			151 TS and 183 controls (Taiwanese)	[64]
			151 TS and 183 controls (Taiwanese)	[64]
		However, other studies showed that DRD2 (TaqIA) had no association or linkage with tic severity and TS.	124 Canadian and 48 Oregon (Canadian; Oregon)	[65]
			4 families (17 individuals using a derivative of YGTSS; 47 using the TS symptomatology evaluation) (North American)	[66]
			One pedigree, 85 interviewed in 116 members (NA)	[58]
			110 trios (French Canadian)	[47]
			61 TS and 109 parents (Germany)	[67]
**DRD3** (Dopamine receptor D3)	3q13.3	Both family and case-control studies did not find association between DRD3 (Mscl, rs6280) and TS.	465 parent–child trios (NA)	[68]
			One pedigree, 85 interviewed in 116 members (NA)	[58]
			110 trios (French Canadian)	[47]
			160 TS and 90 controls (Chinese)	[69]
**DRD4** (Dopamine receptor D4)	11p15.5	The higher number of DRD4 48 bp VNTR was found to be associated with both TS and OCD. (*p* = 0.004–0.03)	64 family trios (Canadian, Michigan, Oregon)	[70]
			61 OCD with and without tic (Mexican)	[71]
			110 trios (French Canadian)	[47]
		No association was found between 48 bp VNTR and TS.	103 trios and 284 controls (European (Hungarian))	[72]
			5 families (NA)	[73]
			102 trios (NA)	[74]
			266 TS and 236 controls (European (white, non-Hispanic))	[75]
**DRD5** (Dopamine receptor D5)	4p16	Family studies did not find linkage between DRD5 and TS. (LOD < −4)	One pedigree, 85 interviewed in 116 members (NA)	[58]
			106 individuals from 5 families (Canadian)	[76]
**TH** (Tyrosine hydroxylase)	11p15.5	Family studies did not find linkage between TH (STR) and TS. (LOD < −7)	One pedigree, 85 interviewed in 116 members (NA)	[58]
			5 families (NA)	[73]
**DAT1** (Dopamine transporter)	5p15.32	DAT1 40 bp VNTR was associated with both tic severity and tics; whereas D*deI* polymorphism was only associated with TS.	103 trios and 284 controls (European (Hungarian))	[72]
			225 TS and 67 controls (European)	[63]
			266 TS and 236 controls (European (white, non-Hispanic))	[75]
		However, some studies showed there was no linkage between 40 bp VNTR and TS, although there was a trend of excess transmission of allele 10 of VNTR.	266 TS and 236 controls (European (white, non-Hispanic))	[75]
			110 trios (French Canadian)	[47]
			Four extend families (Canadian, Oregon)	[77]
			465 parent–child trios (NA)	[68]
**DBH** (Dopamine β-hydroxylase)	9q34.3	Taq B1 allele was associated with TS. (*p* = 0.012)	352 TS and 148 controls (Non-Hispanic Caucasian)	[63]
		Multiple markers of DBH (TaqI, 19 bp ins/del, (CA)n) have no association or linkage with TS.	One pedigree, 85 interviewed in 116 members (NA)	[58]
			71 nuclear families and 5 large-multigenerational families (Canadian, Turkish)	[78]
			266 TS and 236 controls (European (white, non-Hispanic))	[75]
**COMT** (Catechol-*O*-methyltransferase)	22q11.2	No association between COMT Val158Met and TS.	103 trios and 284 controls (Caucasian)	[72]
			52 TS and 63 controls (Italian)	[79]
			465 parent–child trios (NA)	[68]
**Serotonergic**				
**HTR1A** (Serotonin receptor 1A)	5q11.2–13	Two missenses were found in two TS patients.	56 TS and 20 controls (Toronto)	[80]
		No association was found between Ile-28-Val substitution and TS.	A large pedigree (British)	[58]
			92 TS and 210 controls (Germany)	[81]
**HTR2A** (Serotonin receptor 2A)	13q14–21	HTR2A 102 T/C was associated with Chinese TS. (*p* = 0.02)	157 trios and 120 controls (Chinese)	[82]
**HTR2C** (Serotonin receptor 2C)	Xq22–23	Both rs3813929 and rs518147 polymorphisms were associated with TS. (*p* = 0.01 and 0.02, respectively)	87 TS and 311 controls (European)	[83]
		No association was found between HTR2C and TS	465 parent–child trios (NA)	[68]
**5-HT3A, 3B** (Serotonin receptor 3A, 3B)	11q23.2	No association was found between 5-HT3 (Exon1, 3, 6, 7, 9 at 5-HT3A; Exon5, 6; Intron 4, 6 at 5-HT3B) and TS. (*p* = 0.058–0.098)	49 TS and controls (Germany)	[84]
**5-HT7** (Serotonin receptor 7)	10q23	No genomewide significant linkage was found. (LOD = 2.23)	Single extended pedigree (NA)	[85]
**SERT (SLC6A4)** (5-HT transporter (Solute carrier family 6 members 4))	17q11.2	Common and rare alleles of SLC6A4 were positively associated with TS. (*p* < 0.01)	151 TS and 858 controls (European)	[86]
		No association was found.	52 TS and 63 controls (Italian)	[79]
**TPH2** (Tryptphan hydroxylase 2)	12q21	Positive results were found between TPH (SNP at intron 2) and TS. (*p* = 0.002)	98 TS and 178 controls (Germany)	[87]
		No association was found.	465 parent–child trios (NA)	[68]
**Glutamatergic**				
**EAAT1 (SLC1A3)** (Solute carrier family 1(glial high affinity glutamate transporter, member 3))	5p13.2	A missense (E219D) in EAAT1 was found associated with TS patients. (*p* = 0.009)	256 TS and 224 controls (NA)	[88]
**SAPAP3/DLGAP3** (SAP90/PSD95-associated protein 3)	1p34.3	Some SNPs was found to associated with TS. (*p* = 0.013–0.026)	289 TS trios (USA, Canada, Great Britain and the Netherlands)	[89]
**Histaminergic**				
**HDC** (Histidine decarboxylase)	15q21–22	Family studies showed that rare coding mutation was associated with TS.	520 TS families (European)	[90]
			One large family (NA)	[52]
		No association was found.	465 parent–child trios (NA)	[68]
**HRH3** (Histamine receptor H3)	20	No association was found.	465 parent–child trios (NA)	[68]
**Adrenergic and other neurotransmitters**				
**ADRA1C** (Adrenergic receptor a1C)	8p11.2	Adrenergic receptor 1C (PstI) showed no association with TS.	113 nuclear families (Canadian and Turkish)	[91]
**ADRA2A** (Adrenergic receptor a2A)	10q24–26	Adrenergic receptor 2A (MspI, StyI, (CA)n) showed no association with TS.	113 nuclear families (Canadian and Turkish)	[91]
			160 TS and 83 controls (Taiwanese)	[92]
**ADORA1** (Adenosine A1 receptor)	1q32.1	rs2228079 in exron2 was found associated with TS. (*p* = 0.011)	162 TS and 210 controls (European (Polish))	[93]
**ADORA2A** (Adenosine A2A receptor)	22q11.2	rs5751876 in exron3 was found associated with TS. (*p* = 0.017)	162 TS and 210 controls (European (Polish))	[93]
**MAO-A** (Monoamine oxidase A)	Xp11.3	VNTR in MAO-A was found associated with TS. (*p* < 0.05)	110 trios (French Canadian)	[47]
			229 TS and 90 controls (European (non-Hispanic Caucasians))	[94]
		No association was found	465 parent–child trios (NA)	[68]

NA: ethnicity information not available; LOD: logarithm of the odds (to the base of 10); SNPs: single nucleotide polymorphisms; YGTSS: Yale Global Tic Severity Scale; VNTR: variable number tandem repeat; ADHD: attention deficit hyperactivity disorder; OCD: obsessive-compulsive disorder.

**Table 2:** Genome-wide association studies (GWAS) findings in main psychiatric disorders.

Diseases	Case/Control D: Discover; R: Replication; C: Combined	Ethnicity	Analysis	Results	No. of Loci	No. of Genes	Top Candidate Genes	Top SNP Chromosomal Location	*p*-Value of Top SNP; Odds Ratio with Confidence Interval	Reference
**SCZ**	D:34,241/45,604/1235 trios R: 1513/66,236 C: 36,989/113,075	European/East Asian	Meta-analysis	128 independent associations spanning 108 conservatively defined loci that meet genome-wide significance (*p* ≤ 5 × 10^−8^), 83 of which have not been previously reported.	108	348	MHC region, DRD2, GRM3, GRIN2A, SRR, GRIA1, CACNA1C, CACNB2, CACNA1I	rs115329265; 6p22.1	3.48 × 10^−31^; OR: 1.205 (95% CI: 1.168–1.244)	[96]
**BPD**	D: 7647/27,303 R: 2137/3168 C: 9784/30,471	European	Meta-analysis	Six autosomal loci exceeded genome-wide significance.	6	7	ERBB2, ELAVL2, MAD1L1, TRANK1, MIR2113, POU3F2, DDN	rs9834970; 3p22.2	4.83 × 10^−10^; OR: 0.88 (95% CI: 0.85–0.92)	[115]
**MDD**	D: 8920/9519 R: 13,238/124,230 C: 22,158/133,749	European	Meta-analysis	Identified one replicated genome-wide significant locus associated with adult-onset (>27 years) MDD.	1	7	C3orf70, VPS8, EHHADH, MAP3K13, C3orf70, VPS8, MAP3K13	rs7647854; 3q27.2	5.2 × 10^−11^; OR: 1.16 (95% CI: 1.11–1.21)	[116]
**ASD**	D: 7387/8567 R: 9152/148,667 C: 16,539/157,234	European	Meta-analysis	None of the markers investigated exceeded genomewide threshold in the discovery cohort; meta-analyzed against the Danish iPSYCH data a single GWSA; cross-disorder ASD and schizophrenia meta-analyses identified 12 GWS loci not previously identified as GWS in the PGC schizophrenia GWAS.	1	13	C10orf76, CUEDC2, ELOVL3, FBXL15, GBF1, HPS6, LDB1, MIR146B, NFKB2, NOLC1, PITX3, PPRC1, PSD	rs1409313 (meta-analyzed against the Danish iPSYCH data a single GWAS association); 10q24.32	1.058 × 10^−8^; OR: 1.12 (95% CI: 1.08–1.16)	[117]
**Cross-disorder**	D: 6990 BPD, 9227 MDD, 9379 SCZ, 161 ASD, 4788 ASD trios, 840 ADHD, 1947 ADHD trios/27,888 R: NA C: 33,332/27,888	European	Cross-disorder analysis	SNPs at four loci surpassed the cutoff for genome-wide significance (*p* ≤ 5 × 10^−8^) in the primary analysis: regions on chromosomes 3p21 and 10q24, and SNPs within two L-type voltage-gated calcium channel subunits, CACNA1C and CACNB2.	4	4+	ITIH3, AS3MT, CACNA1C, CACNB2	rs2535629; 3p21.1	2.54 × 10^−12^; OR: 1 × 10 (95% CI: 1.07–1.12)	[118]
**TS**	D: 1285/4964 R: 211/285 C: 1496/5249	European/Ashkenazi Jews/French Canadians/Latin American	Meta-analysis	One marker achieved a genome-wide threshold of significance (*p* ≤ 5 × 10^−8^).	1	1	COL27A1	rs7868992; 9q32	2.94 × 10^−8^;	[110]
	D: 609/610 R: 1285/4964 C: 1894/5574	European, Hungary, Germany, Austria, Italy, Greece, French Canadian	Meta-analysis	No markers achieved a genome-wide threshold of significance (*p* ≤ 5 × 10^−8^).	0	0	NTN4	rs2060546; 12q22	5.80 × 10^−7^;	[111]
	D: 1310 OCD, 834 TS, 579 TS + OCD/5667/290 OCD trios R: NA C: 2763/5667/290 trios	European, South African Afrikaner, Ashkenazi Jewish	Cross-disorder analysis	No individual single-nucleotide polymorphisms (SNPs) achieved genome-wide significance; the GWAS signals were enriched for SNPs strongly associated with variations in brain gene expression levels (expression quantitative loci, or eQTLs); No significant polygenic signal was detected across the two disorders.	0	0	POU1F1, CHMP2B, MIR4795	rs4988462; 3p11	3.70 × 10^−7^;	[112]
**OCD**	D: 1406 OCD, 1 489 controls from 1065 families; 192/1984 R: NA C: 1598/3474	NR (U.S.)	Discovered-analysis	Identified interesting candidate genes for OCD, but failed to detect any genome-wide significant finding.	0	0	PTPRD, NEUROD6, SV2A, GRIA4, SLC1A2	rs4401971; 9p23	4.13 × 10^−7^	[104]
	D: 1465/5557/400 trios R: NA C: 1465/5557/400 trios	European, NR, Hispanic or Latin American (U.S., United Arab Emirates, Brazil, Italy, Netherlands, Canada, South Africa, Germany, Costa Rica, France, Mexico)	Meta-analysis	No SNP was identified to be associated with OCD at a genome-wide significant level in the combined trio-case-control sample; enrichment of methylation QTLs (*p* < 0.001) and frontal lobe expression quantitative trait loci (eQTLs) (*p* = 0.001) was observed within the top-ranked SNPs (*p* < 0.01) from the trio case-control analysis.	0	0	FAIM2	rs297941; 12q13.12	4.13 × 10^−7^;	[105]
**ADHD**	D: 17,666 children R: NA C: 17,666 children	NR (U.S., Australia, NR)	Meta-analysis	Meta-analysis did not detect genome-wide significant SNPs, but three genes showed a gene-wide significant association (*p* values between 1.46 × 10^−6^ and 2.66 × 10^−6^); SNP-based heritability ranged from 5% to 34%.	0	0	LMOD2, WASL, ASB15	rs56159542 19p13.11	1.48 × 10^−7^	[113]
	D: 896/2455/2064 trios R: NA C: 896/2455/2064 trios	European	Meta-analysis	No genome-wide significant association (*p* ≤ 5 × 10^−8^) was found.	0	0	SHFM1	rs1464807 7q21.3	1.1 × 10^−6^	[114]

D: Discovery, R: Replication, C: Combined; NR: ethnicity not reported; OR: odds ratio; SCZ: schizophrenia; BPD: bipolar disorder; MDD: major depression; ASD: autism spectrum disorder; ADHD: attention-deficit hyperactivity disorder; GWAS: genomewide association study.

**Table 3:** Genomic structural aberrations in TS.

Cytoband	Variation Type	Karyotype	Carrier Frequency and Phenotype	Candidate Region	Candidate Gene	Reference
**6q16**	Translocation and deletion	46, XY, balanced t(6;22)(q16.2;p13)	One family, 2 carriers of translocation + deletion: proband with TS + OCD; mother with OCD	A 400 kb deletion, 1.3 Mb telomeric to the translocation breakpoint, was also identified on 6q16.	GPR63, NDUFA4, and KLHL32 in the 400 kb deletion on 6q16	[127]
**7q22–q31**	Translocation	46, XY, t(7;18)(q22;q22.3)	One pedigree with 12 individuals; 9 carriers: 4 with vocal tics; 1 with motor tics; 1 with TS, 3 without TS	Breakpoint at 7q22 was localized between markers D7S515 and D7S522.	IMMP2L	[45]
	Duplication/Insertion	46, XY, dup(7)(q22.1–q31.1)	1 patient with TS + depression + delayed speech + mental retardation	Breakpoint on chr7 was mapped to 7q22–q31, between D7S515 and D7S552, 6.5 kb.	IMMP2L	[46,128]
	Translocation and deletion	46, XY, t(2;7)(p24.2;q31)del(7)(q31.1q31.2)	1 TS patient with motor tics + language impairment	Translocation breakpoint on chr7 was mapped to 7q31. A 7.25 Mb deletion within introns 2–3 on 7q31.1–31.2 was identified.	IMMP2L	[122]
	Deletion	Intragenic deletion	188 TS patients and 316 controls: 7 out of 188 TS; 3 out of 316 controls carried the deletion (*p* = 0.0047)	49~331 kb deletions were identified at the 5’end of IMMP2L gene.	IMMP2L	[123]
**7q35–q36**	Complex chromosomal insertion/translocation	Father: 46, XY, inv(2)(p23q22), ins(7;2)(q35q36;p21p23); Daughter and son: 46, XX/XY, der(7)ins(7;2)(q35q36;p21p23)	One family with 3 carriers: daughter and son with TS + OCD; father with OCD + depression	a chromosome 2p21–p23 insertion on chromosome 7q35–q36, interrupting the contactin-associated protein 2 gene (CNTNAP2).	CNTNAP2	[124]
	Translocation	Proband and aunt: 46, XY/XX, der(7)t(7;15)(q35;q26.2)	One large family with 5 carriers: 2 (proband and aunt) with multiple congenital malformations, severe mental retardation and did not have any language development, and scoliosis. Father, grandmother, father of grandmother: 46, XY/XX, balanced t(7;15)(q35;q26.2) without TS and other malformations	Breakpoint localized to a region of approximately 21 kb within intron 11 of the CNTNAP2 gene; and carriers without TS.	CNTNAP2	[125]
**8q13–q22**	Translocation	46, XY, t(6;8)(p23;q13)	One family with 3 carriers: proband with TS + OCD + LD; half-sister with TS + OCD; mother with LD + TS?; One family with 1 carrier: with TS + ADHD	Breakpoint within 8q13.	Unknown	[129]
	Translocation	46, XY, t(1;8)(q21.1;q22.1)	4 carriers in one family, 1 without TS; 1 with TS + ADHD + OCD, 2 with motor tics + ADHD	Breakpoint within 8q22.	CBFA2T1 located 11 kb distal to the 8q breakpoint	[130]
**9p**	Deletion	46, XY, del(9)(qter—p2304:)	One patient with TS + OCD + ADD	Breakpoint lies on chr9.	Unknown	[131]
	Translocation	46, XY, t(3;9)(q25.1;q34.3)	1/176 TS cases	Breakpoint on chr9q34.4 within intron7 of OLFM1 gene.	OLFM1	[132]
	Translocation	46, XX/XY, t (3;9) (q25.1;q34.3)	One family with 2 carriers with TS	Unknown	Unknown	[132]
**13q**	Var321, inversion	46, XY, inv(13)(q31.1;q33.1)	1/174 TS patients: with TS+ADHD; 2 patients carried var321	Breakpoint spans the 13q31.1 and 13q33.1.	SLITRK1, ERCC5 and SLC10A2	[51]
	Var321, inversion	46, XY, inv(13)(q31.1;q33.1)	2/174 TS patients carried var321, none of 2148 controls carried var321 (*p* = 0.0056)	Breakpoint spans the 13q31.1 and 13q33.1.	SLITRK1, ERCC5 and SLC10A2	[133]
**15q13.3; Xq21.31**	Microduplication and deletion	Deletion in 15q13.2, duplication in both 15q13.3 and Xq21.31	One family with 2 carriers: proband with TS + OCD + rage attack; mother with ADHD	Breakpoint within 15q13.2, 15q13.3 and Xq21.31 (2-bp deletion at 15q13.2; 433 kb duplication at 15q13.3; 732 kb duplication at Xq21.31).	CHRNA7, PABPC5 and PCDH11X	[134]
**15q13–q22.3**	Inversion	46, XY, inv(15)(q13;q22.3)	One family with 1 carrier: proband with motor tics + ADHD + OCD + development delay	Inversion spans 15q13–q22.	Unknown	[135]
**16q22–q23**	Fragile sites	46, XX/XY, fr(16)(q22–23)	3/281 carriers: one with TS + Huntington’s Disease; one with TS + BPD + ASD + MR; one with TS + BPD + MR	Fragile site lies within 16q22–q23.	Unknown	[136]
**17p11**	Translocation	46, XY, t(6;17)(q21;p11)	One family with 2 carriers: proband with TS; son with TS + LD	Breakpoint lies within 17p11.	Unknown	[137]
	Deletion	46, XY, del(17)(p11.2)	One patient with TS + SMS + SIB + ADHD + OCB		Unknown	[138]
**18q21.1–q22.3**	Translocation	46, XX, t(2;18)(p12;q22)	One patient with OCD	Breakpoint lies within 18q.	CDH7 and CDH19	[139]
**18q21.1–q22.2**	Inversion	46, XY, inv(18)(q21;q22)	One patient with tics + OCD	Breakpoint lies at 18q, the inverted chromosome showing delayed replication timing.	2 transcripts, GTSCR-1 and CIS4	[140]
**22q11**	Deletion	46, XX, del(22)(q11)	One family with 2 carriers: proband with TS + ADHD + OCD + MR; mother with phonic tic	A deletion at 22q11.	Unknown	[141]
**Xp22.3**	Deletion	46, XY	One family with 3 carriers: proband with TS + ASD; brother with TS + ADHD, mother without TS, but with depression + anxiety + learning disability	A small deletion encompassing exons 4, 5, and 6 of NLGN4 at Xp22.3.	NLGN4	[126]

LD: learning disorder; ADD: attention deficit disorder; MR: mental retardation; SMS: Smith-Magenis syndrome; SIB: self injurious behavior; OCB: obsessive compulsive behavior.

**Table 4:** Copy number variations in TS.

Reference	Sample Size Case/Controls	Ethnicity	Genotyping Technology	Targeted CNV	Genomewide Finding	Candidate Region	Type of CNV	Frequency	CNV Size	Candidate Gene	CNV Type
**Sundaram et al., 2010 [152]**	111/73	European American	Genomewide SNP chip genotyping	Recurrent or de novo rare exonic CNVs	5 rare CNVs were found in 10 out of 111 patients with TS.	3q25.1	Deletion	3/111 in cases, 0/73 in controls	30–40 kb	AADAC	Recurrent
						2p16	Deletion	2/111 in cases, 0/73 in controls	230–270 kb	NRXN1	Recurrent
						10q21	Deletion	2/111 in cases, 0/73 in controls	90–180 kb	CTNNA3	Recurrent
						Chr14	Deletion	2/111 in cases, 0/73 in controls	400–500 kb	FSCB	Recurrent
						Chr21	Duplication	1/111 in cases, 0/73 in controls	170–180 kb	KCNE1-KCNE2-RCAN1	*De novo*
**Fernandez et al., 2012 [150]**	460 (148 trios)/1131 (436 trios)	European	Genomewide SNP genotyping	Rare CNVs (<1%)	1. No significant increase in the number of de novo or transmitted rare CNVs in cases versus controls; 2. Enrichment of genes within histamine receptor (subtypes 1 and 2) signaling pathways by pathways analyses.	Chr5	Duplication		51.8 Mb	447 RefSeq	*De novo*
						6p25.3	Duplication		316 kb	1 RefSeq gene	*De novo*
						20p13	Deletion		1.2 Mb	27 RefSeq genes	*De novo*
						22q11.21	Duplication		2.5 Mb	56 RefSeq genes	*De novo*
**Nag et al., 2013 [153]**	232/234	Latin American	Genomewide SNP genotyping	Large CNVs	1. The rearrangements of COL8A1 and NRXN1 have a nominal significance; 2. Cases with higher large CNV burden: 25/232 in TS; 15/234 in controls (*p* = 0.006).	3q12.1	Duplication	7/232 in cases, 0/234 in controls *p* = 0.004	~600 kb	COL8A1	*De novo*
						2p16	Deletion	4/232 in cases, 0/234 in controls *p* = 0.03	~400 kb	NRXN1	*De novo*
**McGrath et al., 2014 [154]**	1086 TS, 1613 OCD/1789	European	Genomewide SNP genotyping	Large, rare CNVs	Burden of large deletions CNVs previously associated with other neurodevelopmental disorders increased 3.3-fold, whereas no global significant difference in burden of large rare CNVs (*p* = 0.09).	16p13.11	Deletion	5 cases deletions in 16p13.11 locus, 3 deletions are de novo	>500 kb		*De novo*
**Bertelsen et al., 2016 [155]**	1181/118,730	European	qPCR or genome-wide genotyping	AADAC deletion	AADAC deletion association test: *p* = 4.6 × 10^−4^; OR = 2.1; 95% CI (1.37–3.07).	3q25.1	Deletion	43/1181 (1.82%) in TS cases, 2340/118,730 (0.99%) in controls	~36 kb	AADAC	Recurrent
**Huang et al., 2017 [156]**	2434/4093	European	Genomewide SNP genotyping	Rare CNVs ≥ 30 kb	1. Enrichment of global CNV burden, for large (>1 Mb), singleton events (OR = 2.28, 95% CI (1.39–3.79), *p* = 1.2 × 10^−3^); 2. Enrichment of global CNV burden of known, pathogenic CNVs (OR = 3.03,95% CI (1.85–5.07), *p* = 1.5 × 10^−5^); 3. Genome-wide significant loci: (NRXN1 deletions, OR = 20.3, 95% CI (2.6–156.2); CNTN6 duplications, OR = 10.1, 95% CI (2.3–45.4)).	3p26	Duplication	12/2434 (0.49%) in cases, 2/4093 (0.05%) in controls	~640 kb	CNTN6	Recurrent
						2p16	Deletion	12/2434 (0.49%) in cases, 1/4093 (0.02%) in controls		NRXN1	

CNV: copy number variation.

**Table 5:** Main whole exome sequencing/whole genome sequencing (WES/WGS) findings in major psychiatric disorders.

Diseases	Technology	Study Design	Sample Size	Project	Finding	Year	Reference
**ASD**	WES	Case-control	3871 ASD cases and 9937 controls (15,480 DNA samples)	Autism Sequencing Consortium (ASC)	33 ASD risk genes, and rare coding variations enriched in 107 genes implicated in synaptic, transcriptional, and chromatin remodeling pathways; de novo loss-of-function mutations in over 5% of autistic subjects.	2014	[166]
**ASD**	WES	Family study	2517 families (2508 probands, 1911 siblings, 5034 parents)	Simons Simplex Collection (SSC)	27 ASD associated genes; 13% of de novo (DN) missense mutations and 42% of DN likely gene-disrupting (LGD) mutations contributed to 12% and 9% of diagnoses, respectively. Including copy number variants, coding DN mutations contribute to about 30% of all simplex and 45% of female diagnoses.	2014	[167]
**ASD**	SNP chip and WES	Family study	2591 families (10,220 individuals)	SSC, ASC	Small de novo deletions overlaps high effect with de novo loss of function; Identified 71 ASD risk loci, including 6 CNV regions and 65 risk genes.	2015	[168]
**ASD**	WES	Family study	5947 families (4032 trios, 1918 quads)	SSC, ASC	Identified 7.5% of de novo mutations as postzygotic mosaic mutations (PZMs); Damaging, nonsynonymous PZMs within critical exons of prenatally expressed genes were more common in ASD probands than controls (*p* < 1 × 10^−6^), and genes carrying these PZMs were enriched for expression in the amygdala (*p* = 5.4 × 10^−3^).	2017	[169]
**SCZ**	WES	Case-control	2536 SCZ and 2543 controls	Swedish SCZ case-control study via Hospital Discharge Register	Polygenic burden of SCZ primarily arising from rare disruptive mutations distributed across many genes and enrichment in the voltage-gated calcium ion channel and the signaling complex formed by the activity-regulated cytoskeleton-associated (ARC) scaffold protein of the postsynaptic density (PSD).	2014	[170]
**SCZ**	WES	Combined family and case-control study	4264 cases, 9343 controls and 1077 trios	UK10K schizophrenia analysis (UK, Finnish), Swedish schizophrenia case-control study	Histone H3K4 methylation pathway is associated with SCZ; Genome-wide significant association between rare loss-of-function (LoF) variants in SETD1A and risk for schizophrenia (*p* = 3.3 × 10^−9^).	2016	[171]
**SCZ**	WES	Case-control	4946 SCZ, 6242 controls, and 1144 with other psychiatric illnesses	Swedish SCZ case-control study via Hospital Discharge Register	Ultra rare gene-disruptive and putatively protein-damaging variants were more abundant in schizophrenia cases than controls (*p* = 1.3 × 10^−10^).	2016	[172]
**SCZ**	WES	Meta-analysis for combined SNVs and CNVs	4133 SCZ and 9274 controls (4,133 SCZ and 9274 controls AND 1077 trios; 6882 cases and 11,255 controls using CNVs)	UK10K, INTERVAL, Finnish SCZ STUDY, Swedish SCZ Study	Rare, damaging variants contribute to the risk of schizophrenia both with and without intellectual disability; and support an overlap of genetic risk between schizophrenia and other neurodevelopmental disorders.	2017	[173]
**BPD**	WES	Combined family and case-control study	8 families (36 BPD), independent case-control samples consisting of 3541 BPD cases and 4774 controls	Swedish Exome Sequencing Study; BRIDGES	84 rare (frequency < 1%), damaging variants segregating within families; the case-control meta-analyses yielded 19 genes that were nominally associated with BPD; overlap of potential risk genes with autism and schizophrenia.	2016	[174]
**BPD**	WES		4 families (15 individuals)	NIMH Bipolar Genetics Initiativee	14 variants in 14 genes were associated with bipolar disorder when tested against 2545 unaffected controls and 2543 patients with schizophrenia (*p* < 0.05 after Bonferroni correction).	2017	[175]
**MDD**	WES	Combined family and case-control linkage and association studies	Discovery cohort: 2393 individuals; Replication cohort: 1604 individuals	Discovery: the Erasmus Rucphen Family (ERF) study for depressive symptoms; Replication: Rotterdam Study for depressive symptoms	Missense c.1114C >T mutation (rs115482041) in the RCL1 gene segregating with depression across multiple generations. Rs115482041 showed significant association with depressive symptoms (N = 2393, βT-allele = 2.33, *p*-value = 1 × 10^−4^) and explained 2.9% of the estimated genetic variance of depressive symptoms (22%) in ERF; and significant association with depressive symptoms in samples from the independent population-based Rotterdam study (N = 1604, βT-allele = 3.60, *p*-value = 3 × 10^−2^).	2017	[176]
**MDD**	WES	Combined family and case-control study	Discovery cohort: 1265 individuals Replication cohort: 3612 individuals	Discovery cohort: Rotterdam Study for depressive symptoms; Replication cohort: Erasmus Rucphen Family (ERF) study	A missense Asn396Ser mutation (rs77960347) in the endothelial lipase (LIPG) gene, occurring with an allele frequency of 1% in the general population, which was significantly associated with depressive symptoms (*p*-value = 5.2 × 10^−8^, β = 7.2). Replication in three independent data sets (N = 3612) confirmed the association of Asn396Ser (*p*-value = 7.1 × 10^−3^, β = 2.55) with depressive symptoms.	2017	[177]
**MDD**	WES	Combined family and case-control study	Discovery: 1999 individuals; Replication: 2356 individuals	Discovery: the Erasmus Rucphen Family (ERF) study for depressive symptoms; Replication: Rotterdam Study for depressive symptoms	Rare nonsynonymous variants in NKPD1 is associated with depressive symptoms in discovery cohort (*p* = 3.7 × 10^−8^); variants explained 0.9% of the age- and sex-adjusted variance and 3.8% of heritability of depressive symptoms in the ERF population; meta-analysis of the discovery and replication studies improved the association signal (*p* = 1.0 × 10^−9^).	2017	[178]
**ASD**	WGS	Family study	2626 ASD cases and 2579 family controls	AGRE, autism Treatment Network, Genomes to Outcomes Study; Baby Siblings Research Consortium, The Autism Simplex Collection, Infant Sibling Study, Pathways in ASD	An average of 73.8 de novo SNVs and 12.6 de novo insertions and deletions or CNVs per ASD subject; 18 new candidate ASD-risk genes were identified. In 294 of 2620 (11.2%) of ASD cases, a molecular basis could be determined and 7.2% of these carried copy number variations and/or chromosomal abnormalities, emphasizing the importance of detecting all forms of genetic variation as diagnostic and therapeutic targets in ASD.	2017	[179]
**BPD**	WGS	Family study	41 families (200 individuals) and 254 individuals as controls	NIMH	An increased burden of rare variants in genes encoding neuronal ion channels, including subunits of GABAA receptors and voltage-gated calcium channels; most of the risk variants in noncoding predicted regulatory effects.	2015	[180]
**SCZ**	WGS	Family study	9 multiplex families (90 individuals)	Coriell Institute in Camden	In one family, seven siblings with schizophrenia spectrum disorders each carry a novel private missense variant within the SHANK2 gene. In another family, four affected siblings and their unaffected mother each carry a novel private missense variant in the SMARCA1 gene on the X chromosome.	2016	[181]
**MDD**	WGS	Case-control, low coverage WGS	Discovery: 5303 cases, 5337 controls (Chinese women); Replication cohort 1: a separate Han Chinese cohort of 3231 cases with recurrent MDD, and 3186 controls; Replication cohort 2: 9240 European MDD cases and 9519 controls	CONVERGE Consortium	Two genome-wide significant loci contributing to risk of MDD on chromosome 10: SIRT1 gene (*p* = 2.53 × 10^−10^) and LHPP gene (*p* = 6.45 × 10^−12^); common SNPs explained between 20% and 29% of the variance in MDD risk; support a substantial polygenic component to the risk of MDD involving many alleles of individually very small effect; the MDD risk allele frequencies of rs12415800 and rs35936514 are different according to ethnicity, confirmed in Chinese replication cohort but failed in European MDD cohorts. Results support a complex etiology for MDD.	2015–2017	[182,183,184,185]
